# Treatment and outcome of hepatorenal syndrome in Japan: a retrospective cohort study using a national inpatient database

**DOI:** 10.1186/s12876-023-02858-5

**Published:** 2023-06-23

**Authors:** Kazuya Okushin, Hayato Yamana, Ryosuke Tateishi, Masaya Sato, Takeya Tsutsumi, Hiroki Matsui, Kiyohide Fushimi, Hideo Yasunaga, Kazuhiko Koike, Mitsuhiro Fujishiro

**Affiliations:** 1 Department of Gastroenterology, Graduate School of Medicine, The University of Tokyo, 7-3-1 Hongo, Bunkyo-ku, Tokyo, 113-8655 Japan; 2 Department of Infection Control and Prevention, Graduate School of Medicine, The University of Tokyo, Tokyo, Japan; 3 Data Science Center, Jichi Medical University, Shimotsuke, Japan; 4 Department of Clinical Epidemiology and Health Economics, School of Public Health, The University of Tokyo, Tokyo, Japan; 5 Department of Health Policy and Informatics, Tokyo Medical and Dental University Graduate School, Tokyo, Japan; 6 Kanto Central Hospital, Tokyo, Japan

**Keywords:** Hepatorenal syndrome, Liver cirrhosis, Renal insufficiency

## Abstract

**Background:**

Hepatorenal syndrome (HRS) is a life-threatening complication of end-stage liver disease. This study aimed to clarify the status of HRS in Japan by analyzing the Japanese Diagnosis Procedure Combination database.

**Methods:**

Patients hospitalized for cirrhosis and HRS from July 2010 to March 2019 were sampled. They were divided into two groups according to their prognosis upon discharge: the transplant-free survival group and the death or liver transplantation group. The two groups’ baseline patient characteristics and treatments were compared.

**Results:**

The mean age of the 1,412 participants was 67.3 years (standard deviation: 12.3 years), and 65.4% were male. The Child–Pugh grades was B and C in 18.8% and 81.2%, respectively. Hepatocellular carcinoma was present in 27.1% of the patients, and the proportion of spontaneous bacterial peritonitis was 2.3%. Albumin, noradrenaline, and dopamine were administered to 57.9%, 8.0%, and 14.9% of the patients, respectively; 7.0% of the patients underwent renal replacement therapy; and 5.0% were admitted to the intensive care unit. Intravenous antibiotics were administered to 30.8% of the patients. A total of 925 patients (65.5%) died or underwent liver transplantation. In addition to a higher proportion of patients with poor baseline liver function, the death or liver transplantation group included more males, patients with hepatocellular carcinoma, and those with spontaneous bacterial peritonitis.

**Conclusions:**

HRS in Japan has a high mortality rate. Albumin was administered to over 50% of participants. Although noradrenaline is recommended in Japanese clinical guidelines, dopamine was more frequently used as a vasoconstrictor in clinical practice.

**Supplementary Information:**

The online version contains supplementary material available at 10.1186/s12876-023-02858-5.

## Background

Hepatorenal syndrome (HRS) is a serious complication of end-stage liver disease [1, 2]. HRS had been considered a type of "functional" renal failure without structural changes. However, recent research has recognized the role of systemic inflammation, oxidative stress, and bile salt-related damage in causing abnormalities in the arterial circulation [1, 3, 4]. HRS is associated with high mortality [5, 6], and long-term survival can only be achieved with liver transplantation [7, 8].

Vasoconstrictors and volume expansion with albumin infusion are recommended treatment options for HRS [9–11]. Among vasoconstrictors, noradrenaline is recommended in Japanese clinical guidelines [9, 10], whereas terlipressin, a vasopressin analog, is commonly used in other countries [12–15]. Reports of noradrenaline for HRS suggest its efficacy, cost-effectiveness, and non-inferiority to terlipressin [16–18]. However, there have been no reports in Japan wherein noradrenaline is recommended. Furthermore, although there have been some single-center experiences of HRS after liver transplantation [19–21], a nationwide study of the clinical characteristics, treatment details, and mortality of HRS has not been conducted.

To clarify the current status of HRS, we conducted a descriptive study using a national Japanese inpatient database.

## Methods

### Data source

The Japanese Diagnosis Procedure Combination database is a nationwide administrative database of claims and discharges abstract data [22, 23]. The number of participating hospitals exceeds 1,000, covering approximately 90% of tertiary care hospitals in Japan and comprising data from approximately seven million inpatients per annum [24]. A validation study showed high sensitivity and specificity of the data, particularly for the recorded procedures [22]. The database contains unique hospital identifiers; patient baseline characteristics, including age, sex, height, and weight; primary diagnosis, comorbidities upon admission, and in-hospital complications recorded using the International Classification of Diseases, 10th revision (ICD-10) codes and text data in Japanese; admission and discharge status, including an indicator of whether a patient died of the primary diagnosis or not; surgical and non-surgical procedures; drugs and devices used; and disease severity. Recording each patient’s Child–Pugh score (encephalopathy, ascites, bilirubin, albumin, and international normalized ratio of prothrombin time) is mandatory for patients with liver cirrhosis.

The current study was performed in accordance with the Declaration of Helsinki. The study was approved by the Institutional Review Board of the Graduate School of Medicine, The University of Tokyo (No. 3501). The need to obtain informed consent was dispensed by the Institutional Review Board of the Graduate School of Medicine, The University of Tokyo due to the anonymous nature of the data.

### Study design and population

Using the Diagnosis Procedure Combination database, we extracted data on patients admitted for HRS from July 1, 2010, to March 31, 2019. Patients diagnosed with liver cirrhosis from any cause (ICD-10 codes, K70.2, K70.3, K71.7, or K74) at the time of admission, and HRS (ICD-10 code, K76.7) during the hospitalization period, were eligible. We excluded patients < 20 years of age and those lacking Child–Pugh scores. We further excluded patients with Child–Pugh class A cirrhosis [25] or acute kidney failure (ICD-10 code, N17).

### Study variables

We obtained the following data: sex, age, body mass index, Child–Pugh score, hepatocellular carcinoma complications (ICD-10 codes, C22.0), spontaneous bacterial peritonitis (SBP) complications (retrieved from diagnostic names registered in Japanese), and treatment details including liver transplantation, albumin infusion, use of vasopressor drugs, intensive care unit (ICU) admission, renal replacement therapy, intravenous antibiotics, and plasma exchange. Renal replacement therapy included hemodialysis and continuous hemodiafiltration. The intravenous antibiotics included carbapenems, quinolones, cephems, penicillins, and anti-methicillin-resistant *Staphylococcus aureus* agents. Etiology of liver cirrhosis was defined as follows: hepatitis B virus (ICD-10 codes, B16.2, B16.9, and B18.1), hepatitis C virus (ICD-10 code, B18.2), alcohol (ICD-10 codes, K70.2 and K70.3), and unspecified.

### Statistical analysis

Descriptive statistics were expressed as mean with standard deviation or median with 25th and 75th percentiles for continuous variables and numbers with percentages for categorical variables. We divided the participants according to (1) those who survived without liver transplantation and (2) those who died or underwent liver transplantation, as per their discharge status. The baseline characteristics of the two groups were compared. The chi-squared test was used to compare categorical variables, and the *t*-test and Wilcoxon rank-sum test were used to compare continuous variables. All statistical tests were conducted at a two-sided significance level of 0.05. We also examined the treatments provided during hospitalization and the day of hospitalization on which each treatment was initiated in the two groups. Analyses were performed using the Stata version 16 software (StataCorp, College Station, TX, USA).

## Results

### Study population

**Fig. 1:**
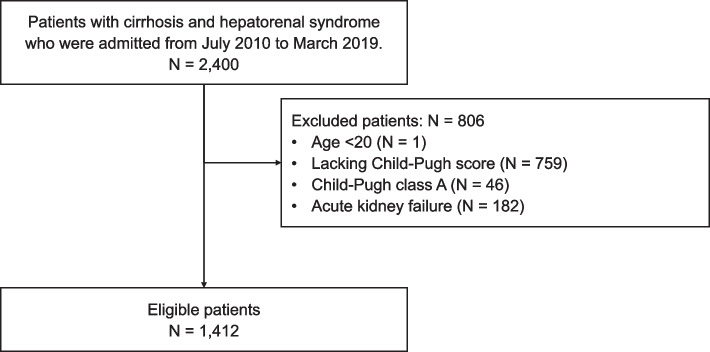
Patient selection flowchart

Among cirrhosis patients diagnosed with HRS during hospitalization (*n* = 2,400), we excluded one patient aged < 20 years and 759 patients lacking Child–Pugh scores. We further excluded 46 patients with Child–Pugh class A cirrhosis and 182 patients with acute kidney failure. Death or liver transplantation occurred in 622 of the excluded patients (63.0%). The data of the remaining 1,412 patients were analyzed (Fig. 1).

Table 1 presents the baseline patient characteristics. The mean age of all patients was 67.3 years (standard deviation, 12.3 years), and 65.4% were male. The Child–Pugh classes were B and C in 18.8% and 81.2% of the patients, respectively. The etiologies of cirrhosis were hepatitis B virus (2.3%), hepatitis C virus (12.8%), alcohol (33.1%), and unspecified (51.8%). Alcohol was the most frequent etiology in males (43.9%), whereas unspecified was most frequent in females (68.5%) (Supplementary Table 1). Hepatocellular carcinoma was present in 27.1% of the patients, and SBP occurred in 2.3%.

**Table 1:** Baseline characteristics of inpatients with hepatorenal syndrome according to their outcomes at discharge

Variable	Total (*N* = 1,412)	Transplant-free survival (*N* = 487)	Death or liver transplantation (*N* = 925)	*P*-value
Age (year), mean (SD)	67.3 (12.3)	66.6 (12.7)	67.7 (12.1)	0.11
Male sex, n (%)	923 (65.4)	300 (61.6)	623 (67.4)	0.03
Body mass index (kg/m^2^), mean (SD)^a^	24.0 (6.0)	24.3 (5.7)	23.8 (6.2)	0.17
Length of hospital stay (days), median (IQR)	19 (8, 34)	22 (10, 40)	17 (7, 32)	< 0.001
Child–Pugh class, n (%)				< 0.001
B	265 (18.8)	160 (32.9)	105 (11.4)	
C	1,147 (81.2)	327 (67.1)	820 (88.6)	
Child–Pugh score, mean (SD)	11.6 (2.1)	10.6 (1.9)	12.2 (2.0)	< 0.001
Etiology, n (%)				0.24
Hepatitis B virus	32 (2.3)	11 (2.3)	21 (2.3)	
Hepatitis C virus	181 (12.8)	51 (10.5)	130 (14.1)	
Alcohol	468 (33.1)	160 (32.9)	308 (33.3)	
Unspecified	731 (51.8)	265 (54.4)	466 (50.4)	
Hepatocellular carcinoma, n (%)	383 (27.1)	85 (17.5)	298 (32.2)	< 0.001
Spontaneous bacterial peritonitis, n (%)	33 (2.3)	6 (1.2)	27 (2.9)	0.05

*IQR* interquartile range, *SD* standard deviation

^a^Data were missing for 34 patients in the transplant-free group and 100 patients in death or liver transplantation group

Table 2 shows the treatments administered. Albumin, noradrenaline, a combination of albumin and noradrenaline, and dopamine were used in 57.9%, 8.0%, 7.3%, and 14.9% of patients, respectively. Ninety patients (6.4%) received two or more vasoconstrictors. Renal replacement therapy was conducted in 7.0% of the patients, and 5.0% were admitted to the ICU. Intravenous antibiotics were administered to 30.8% of the patients.

**Table 2:** Specific treatment to inpatients with hepatorenal syndrome according to their outcomes at discharge

	Treatment selection, n (%)			Day of treatment initiation, median (interquartile range)		
Variable	Total (*N* = 1,412)	Transplant-free survival (*N* = 487)	Death or liver transplantation (*N* = 925)	Total (*N* = 1,412)	Transplant-free survival (*N* = 487)	Death or liver transplantation (*N* = 925)
Albumin infusion	818 (57.9)	274 (56.3)	544 (58.8)	3 (1, 7)	3 (1, 5)	3 (1, 8)
Noradrenaline	113 (8.0)	20 (4.1)	93 (10.1)	8 (2, 18)	2.5 (1, 10)	9 (3, 21)
Combination of albumin and noradrenaline	103 (7.3)	18 (3.7)	85 (9.2)	2 (1, 6)	1 (1, 2)	2 (1, 7)
Vasopressin	22 (1.6)	4 (0.8)	18 (1.9)	7.5 (2, 14)	4.5 (1.5, 7.5)	10 (2, 15)
Adrenaline	60 (4.2)	7 (1.4)	53 (5.7)	14.5 (3, 30)	12 (1, 42)	15 (3, 29)
Dopamine	210 (14.9)	16 (3.3)	194 (21.0)	8 (2, 21)	4 (1, 9)	9 (2, 22)
Dobutamine	22 (1.6)	0 (0.0)	22 (2.4)	11.5 (3, 36)	-	11.5 (3, 36)
Intensive care unit use	70 (5.0)	14 (2.9)	56 (6.1)	3 (1, 14)	1 (1, 5)	3.5 (1, 18.5)
Renal replacement therapy	99 (7.0)	36 (7.4)	63 (6.8)	9 (2, 21)	4 (2, 13.5)	12 (3, 26)
Hemodialysis	75 (5.3)	31 (6.4)	44 (4.8)	11 (3, 24)	4 (2, 21)	14.5 (5.5, 25.5)
CHDF	36 (2.5)	8 (1.6)	28 (3.0)	8 (2.5, 25.5)	2 (1.5, 5.5)	12 (3.5, 40)
Plasma exchange	9 (0.6)	1 (0.2)	8 (0.9)	6 (2, 13)	2 (2, 2)	8 (2, 14)
Intravenous antibiotics	435 (30.8)	137 (28.1)	298 (32.2)	5 (1, 14)	5 (2, 17)	5 (1, 13)

*CHDF* continuous hemodiafiltration

### Patient characteristics and treatment by status at discharge

Among the 1,412 patients, 918 (65.0%) died without liver transplantation. Seven patients underwent liver transplantation, including one who died post-transplantation. The median time from admission to liver transplantation was 22 days (interquartile range, 14–36 days). Among the 919 deceased patients, the cause of death could be identified in 609 patients.

Frequent causes of death and their ICD-10 codes were liver cirrhosis (K70.3 and K74.6, *n* = 246), hepatocellular carcinoma (C22.0, *n* = 103), hepatic failure (K70.4, K72.0, K72.1, and K72.9, *n* = 100), HRS (K76.7, *n* = 67), and primary biliary cholangitis (K74.3, *n* = 16).

Compared to patients who were discharged alive, those who died or underwent liver transplantation had a larger proportion of males, shorter hospital stays, worse baseline Child–Pugh scores, and a larger proportion of hepatocellular carcinoma (Table 1). Age and body mass index were not associated with the composite outcomes. SBP was more frequently observed in patients who died or underwent liver transplantation. Patients who died or underwent liver transplantation were more likely to receive albumin, noradrenaline, and a combination of both than those who were discharged alive (58.8% vs. 56.3%, 10.1% vs. 4.1%, and 9.2% vs. 3.7%, respectively) (Table 2). Moreover, other vasoconstrictors, including vasopressin and dopamine, were frequently administered to patients who died or underwent liver transplantation. These patients were also more likely to be admitted to the ICU (6.1% vs. 2.9%). Administration of noradrenaline and dopamine, ICU admission, and renal replacement therapy occurred later during the hospitalization period for patients who died or underwent liver transplantation than in those who were discharged alive. The proportion of intravenous antibiotic use was similar between groups.

## Discussion

To the best of our knowledge, this is the first nationwide report on the clinical dynamics of HRS that includes data on treatment details and short-term survival. Over 50% of patients died during hospitalization. Half of the patients received albumin infusion. Noradrenaline was infrequently administered to patients with HRS.

Albumin is recommended as the initial treatment for HRS [9–11]. In addition to its oncotic properties, the antioxidant and anti-inflammatory actions of albumin work to maintain systemic circulation [26, 27]. In this study, half of the patients received albumin during hospitalization.

Terlipressin is widely used as a vasoconstrictor outside Japan. However, terlipressin is not approved in Japan, and therefore noradrenaline is recommended in clinical guidelines [9, 10]. To clarify the treatment selection of vasoconstrictors for HRS, we evaluated noradrenalin, adrenaline, dopamine, dobutamine, and vasopressin, an analog of terlipressin. Noradrenaline and vasopressin were rarely used, while dopamine was the most frequently used medication. The proportion of patients who received vasoconstrictors was higher in those who died or underwent liver transplantation than in those who were discharged alive. Additionally, vasoconstrictors were initiated later, although the length of hospitalization was shorter in this group. These results might reflect the use of these vasoconstrictors during the near-death period. Considering these results and the relatively higher proportion of dopamine, which can be relatively easily administered in the general wards, considerable proportion of patients with HRS in Japan might not be receiving specific and intensive treatment at an appropriate time. This may be due to the fact that liver transplantation for decompensated cirrhosis is conducted on a limited basis due to the scarcity of donors, especially the deceased. There may have been no radical treatment options for HRS other than liver transplantation, and medical treatment is not expected to provide favorable outcomes. However, the updated clinical guidelines have introduced the concept of acute kidney injury into the management of cirrhosis and have provided an early diagnosis and treatment algorithm for HRS [9–11]. The outcomes of HRS may improve in the near future.

We also evaluated infectious complications associated with HRS in this study. Although SBP was infrequently recorded (2.3%), intravenous antibiotics were administered to one-third of the patients. Because we had no information on when HRS and infectious diseases were diagnosed, we could not determine whether the infections triggered HRS. However, given the immunocompromised status of patients with cirrhosis and the frequent use of antibiotics in the present study, infectious diseases including SBP and their management are important in patients with HRS.

Despite these important findings, the study has several limitations. First, the data were retrieved from diagnostic records, as determined by the attending physician. We could not obtain the results of blood examinations, such as creatinine levels; therefore, the disease severity could not be stratified. Additionally, there may be more patients with severe acute kidney injury, particularly HRS type 1, a progressive and fatal type of HRS now referred to as HRS-acute kidney injury [5], among those who died. Second, although we excluded patients with a diagnosis of acute kidney failure, there is a possibility that acute kidney injury, such as acute tubular necrosis, was diagnosed as HRS. Third, the day of initiation of each treatment was recorded, but the timing of HRS diagnosis was unclear. This made it difficult to determine, especially with regard to vasoconstrictors, whether they were administered as a treatment for HRS or for other worsening conditions. Finally, the causal relationship between treatments, such as dopamine or noradrenaline, and prognosis could not be concluded because this study did not adjust for the detailed clinical conditions of the patients.

## Conclusion

In conclusion, this retrospective cohort study using a large-scale national database presented the patient characteristics and therapeutic schedules of patients with HRS in Japan, including their short-term survival. Albumin was administered to over 50% of patients. Although noradrenaline is recommended in Japanese guidelines, dopamine is most frequently used as a vasoconstrictor in clinical practice.

## Supplementary Information

**Additional file 1: Supplementary Table 1. **Baseline characteristics of male and female inpatients with hepatorenal syndrome

## Data Availability

The datasets generated during the current study are not publicly available due to further uses for clinical studies in the future but are available from the corresponding author on reasonable request.

## Abbreviations

HRS: Hepatorenal syndrome
ICD-10: International Classification of Diseases, 10th revision
ICU: Intensive care unit
