# Supplementary material for: Treatment and outcome of hepatorenal syndrome in Japan: a retrospective cohort study using a national inpatient database
Source: BMC Gastroenterol. 2023 Jun 23;23:218. doi: 10.1186/s12876-023-02858-5 (PMC10288750; doi:10.1186/s12876-023-02858-5)
Supplement: Supplementary file 1 — Additional file 1: Supplementary Table 1. Baseline characteristics of male and female inpatients with hepatorenal syndrome [file 12876_2023_2858_MOESM1_ESM.docx]

# **Supplementary Table 1** Baseline characteristics of male and female inpatients with hepatorenal syndrome

| Variables | Total  (*N* = 1,412) | Males  (*N* = 923) | Females  (*N* = 489) | *P*-value |
| --- | --- | --- | --- | --- |
| Age (year), mean (SD) | 67.3 (12.3) | 65.6 (11.7) | 70.6 (12.8) | <0.001 |
| Child-Pugh class, n (%) |  |  |  | 0.009 |
| B | 265 (18.8) | 155 (16.8) | 110 (22.5) |  |
| C | 1,147 (81.2) | 768 (83.2) | 379 (77.5) |  |
| Etiology, n (%) |  |  |  | <0.001 |
| Hepatitis B virus | 32 (2.3) | 24 (2.6) | 8 (1.6) |  |
| Hepatitis C virus | 181 (12.8) | 98 (10.6) | 83 (17.0) |  |
| Alcohol | 468 (33.1) | 405 (43.9) | 63 (12.9) |  |
| Unspecified | 731 (51.8) | 396 (42.9) | 335 (68.5) |  |
